# Imaging language pathways predicts postoperative naming deficits

**DOI:** 10.1136/jnnp.2007.126078

**Published:** 2007-11-15

**Authors:** H W R Powell, G J M Parker, D C Alexander, M R Symms, P A Boulby, G J Barker, P J Thompson, M J Koepp, John S Duncan

**Affiliations:** 1 Department of Clinical and Experimental Epilepsy, Institute of Neurology, University College London, UK; 2 Imaging Science and Biomedical Engineering, University of Manchester, UK; 3 Department of Computer Science, University College London, London, UK; 4 King’s College London, Institute of Psychiatry, Department of Clinical Neurosciences, UK

## Abstract

Naming difficulties are a well recognised, but difficult to predict, complication of anterior temporal lobe resection (ATLR) for refractory epilepsy. We used MR tractography preoperatively to demonstrate the structural connectivity of language areas in patients undergoing dominant hemisphere ATLR. Greater lateralisation of tracts to the dominant hemisphere was associated with greater decline in naming function. We suggest that this method has the potential to predict language deficits in patients undergoing ATLR.

Anterior temporal lobe resection (ATLR) leads to seizure freedom in approximately 60% of patients with refractory temporal lobe epilepsy (TLE), but may be complicated by cognitive impairments. In addition to the well recognised decline in verbal memory, language functions, in particular naming abilities, are impaired following ATLR of the language dominant hemisphere in 30–40% of patients.^1^ ^2^

TLE is often associated with atypical, bilateral or right sided language dominance.^3^ Preoperative functional MRI (fMRI) has been shown to predict language deficits following left ATLR. In a semantic decision making task, fMRI laterality indices were found to be predictive of naming outcome with greater left lateralised language activation in temporal structures being correlated with greater postoperative naming decline.^4^ Devinsky *et al* found that early onset of dominant temporal lobe seizure foci was associated with more widespread or atypical distribution of language areas, particularly naming and reading areas.^5^ Markers of early left hemisphere damage, such as early seizure onset, poor verbal IQ, left handedness and right hemisphere memory dominance, increase the chances of essential language areas being located in more anterior temporal regions. Again, these areas were identified using naming and reading tasks.^6^

Diffusion tensor imaging (DTI) is an MRI technique that evaluates brain structure through the three dimensional measurement of diffusion of water molecules in tissue.^7^ It provides the basis for MR tractography, a method that visualises the trajectories of cerebral white matter tracts non-invasively and rapidly.^8^ We have recently used tractography to study the structural connections underlying functional language regions.^9^ ^10^ These may assist planning surgery to minimise deficits. We report here the use of tractography in patients undergoing ATLR of the language dominant hemisphere, to test the hypothesis that patients with language connections that were more strongly lateralised to the dominant hemisphere would be at greater risk of decline in language function. Given that naming difficulties are the commonest language problems following ATLR, we looked in particular at the connectivity of expressive language regions in the inferior frontal lobe.

## METHODS

### Subjects

We report seven patients (median age 33; range 25–37 years; two females) undergoing dominant ATLR at the National Hospital for Neurology and Neurosurgery for medically refractory TLE. All patients had undergone structural MRI at 1.5 T. Five had hippocampal sclerosis (one of whom additionally had a ganglioglioma in the left fusiform gyrus), one had a medial temporal lobe (MTL) dysembryoblastic neuroepithelial tumour and one a MTL glioma. Video-EEG had confirmed seizures arising from the ipsilateral MTL in all seven. All patients had a normal contralateral hippocampus on qualitative and quantitative MRI. All patients were on antiepileptic medication and all were fluent English speakers. Handedness was determined using a standardised questionnaire. Language dominance was assessed using a range of fMRI tasks,^11^ revealing left hemisphere dominance in all but one patient. This patient was left handed and confirmed to be right hemisphere dominant for language on both fMRI and intracarotid amytal test. His pattern of neuropsychological test results, with a decline in verbal memory score and little change in non-verbal memory, were also in keeping with resection of the language dominant hemisphere. All patients underwent standardised neuropsychological assessment, including the Graded Naming Test,^12^ preoperatively and 3 months following ATLR.

Patient demographics and clinical and neuropsychological data are detailed in table 1. The International League Against Epilepsy classification of postoperative seizure outcome following epilepsy surgery was used.^13^ As previously reported,^9^ we also studied 10 healthy volunteers (median age 29.5; range 23–50 years; six females) with no history of neurological or psychiatric disease. These subjects’ fMRI images were used, along with the patients’, to create the starting points for tractography. The study was approved by the National Hospital for Neurology and Neurosurgery and the Institute of Neurology Joint Research Ethics Committee and informed written consent was obtained from all subjects.

**Table 1:** Patient clinical, demographic and neuropsychological data

Age/sex	Handedness	Epilepsy onset (y)	Seizure types and frequency (per month)	Postop outcome (ILAE class)	MRI and pathological diagnosis	Clinical and EEG	Preop naming (centile)	Postop naming (centile)	Naming change	AEDs (mg/day)	LD
37/M	Left	1	SPS 12 CPS 4	2	Left HS	Left TLE	19 (50^th^)	16 (25^th^)	−3	VPA 800 CBZ 800 LVT 2000	Left
33/M	Right	1	SPS 4 CPS 4	1	Left HS	Left TLE	13 (5^th^)	15 (10^th^)	2	PMD 500 CBZ 1200 CLB 10 TPR 175 LVT 4000	Left
25/F	Right	17	CPS 8 SGTC 0.5	1	Left HS	Left TLE	7 (<1^st^)	5 (<1^st^)	−2	TPR 150 LTG 300	Left
28/M	Right	3	CPS 1	1	Left HS	Left TLE	4 (<1^st^)	7 (<1^st^)	3	LVT 3000 LTG 600	Left
31/M	Right	10	CPS 50 SGTC 3	1	Left MTL DNET	Left TLE	14 (10^th^)	10 (<1^st^)	−4	CBZ 1200 CLN 1.5 LTG 100	Left
37/F	Right	1	SPS 12 CPS 8 SGTC 1	3	Left HS, left fusiform gyrus ganglioglioma	Left TLE	10 (<1^st^)	13 (5^th^)	3	CBZ 1000 CLB 10	Left
36/M	Left	15	SPS 6 CPS 6 SGTC 3	1	Right MTL glioma	Right TLE	17 (25^th^)	2 (<1^st^)	−15	CBZ 1600 CLB 20 LTG 400	Right

AED, antiepileptic drug; CBZ, carbamazepine; CLB, clobazam; CLN, clonazepam; CPS, complex partial seizure; DNET, dysembryoplastic neuroepithelial tumour; HS, hippocampal sclerosis; ILAE, International League Against Epilepsy; LD, language dominance; LTG, lamotrigine; LVT, levetiracetam; MTL, medial temporal lobe; NA, not applicable; PMD, primidone; SGTC, secondary generalised tonic–clonic seizure; SPS, simple partial seizure; TLE, temporal lobe epilepsy; TPR, topiramate; VPA, sodium valproate.

### MRI diffusion tensor imaging

We performed fMRI and DTI for tractography on all patients on a 1.5 T General Electric Signa Horizon scanner (Milwaukee, Wisconsin, USA). The DTI acquisition sequence was a single shot spin-echo echo planar imaging sequence, cardiac gated (triggering occurring every QRS complex), with TE = 95 ms, 96×96 acquisition matrix (128×128 reconstructed) and 22 cm×22 cm field of view. Acquisitions of 60 contiguous 2.3 mm thickness axial slices were obtained, covering the whole brain, with diffusion sensitising gradients applied in each of 54 non-colinear directions (maximum b value of 1148 mm^2^/s (δ = 34 ms, Δ = 40 ms, using full gradient strength of 22 mT/m)) along with six non-diffusion weighted (b = 0) scans. The DTI acquisition time for a total of 3600 images was approximately 25 min (depending on the heart rate).

The diffusion tensor eigen values λ_1_, λ_2,_ λ_3_ and eigen vectors ∊_1,_ ∊_2,_ ∊_3_ were calculated from the DTI data, and fractional anisotropy maps were generated according to the method described by Pierpaoli and colleagues,^7^ using locally written software. We used the method of Parker and Alexander^14^ to avoid ambiguities in voxels containing fibre crossings. Voxels in which the single tensor fitted the data poorly were identified using the spherical–harmonic voxel classification algorithm of Alexander and colleagues.^15^ In these voxels, a mixture of two Gaussian probability densities were fitted and the principal diffusion directions of the two diffusion tensors provided estimates of the orientations of the crossing fibres.^16^ In all other voxels, a single tensor model was fitted.

### Language fMRI tasks

Subjects performed a verb generation task, consisting of a blocked experimental design with 30 s task blocks alternating with 30 s of rest over 5.5 min. During the task, concrete nouns were visually presented every 3 s in blocks of 10 nouns. Subjects were instructed to covertly generate verbs from the nouns during the task block and to silently repeat the nouns during the rest block.^17^ The data were analysed using SPM2 (Wellcome Department of Imaging Neuroscience (http://www.fil.ion.ucl.ac.uk/spm/)). Scans from each subject were realigned using the first as a reference, spatially normalised into standard space and spatially smoothed with a Gaussian kernel of 10 mm FWHM. A two level random effects analysis was used. At the first level, contrast images were produced for each subject, corresponding to the main effects of verb generation against the control condition. At the second level, each subject’s contrast image was entered into a one sample t test to examine the main effect of verb generation across the group.

### Starting points

Maps of the main effect of each task across all subjects (controls and patients combined) were generated, thresholded at p<0.001 (uncorrected) and transformed into each individual’s native space. These reverse normalised group maps were then used to define regions of interest (ROIs) for initiating probabilistic fibre tracking.^10^ Two ROIs were defined in each subject’s native space, one each in the left and right inferior frontal gyrus, by manually drawing over selected areas of fMRI activation on consecutive brain slices, using MRIcro (http://www.psychology.nottingham.ac.uk). The left-sided ROI corresponded to the region of peak activation within the inferior frontal gyrus for verb generation. As no significant activation was seen in this region on the right, a homotopic ROI of identical size was manually defined. All ROIs comprised 125 voxels. 

### Tractography analysis

We used the Probabilistic Index of Connectivity algorithm extended to cope with crossing fibres^14^ ^18^ to track the language related pathways. In this probabilistic tractography algorithm, the streamline process is repeated using Monte Carlo methods to generate maps of connection probability or confidence of connection to chosen start points. Each output connectivity map was spatially normalised and averaged across the group to produce variability maps indicating the degree of spatial variability and overlap of the identified connections. Normalised tract volumes (N(V)) were calculated for the left and right tracts of every subject at a probability threshold of 0.05.^10^ We calculated asymmetry indices (AIs) to assess lateralisation of tracts between hemispheres;


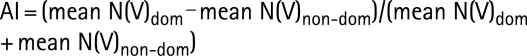


We calculated the Spearman correlation coefficient to test for correlations between tract lateralisation and both preoperative naming score and change in naming score following surgery, and also between tract lateralisation and age of onset of epilepsy.

## RESULTS

**Figure 1:**
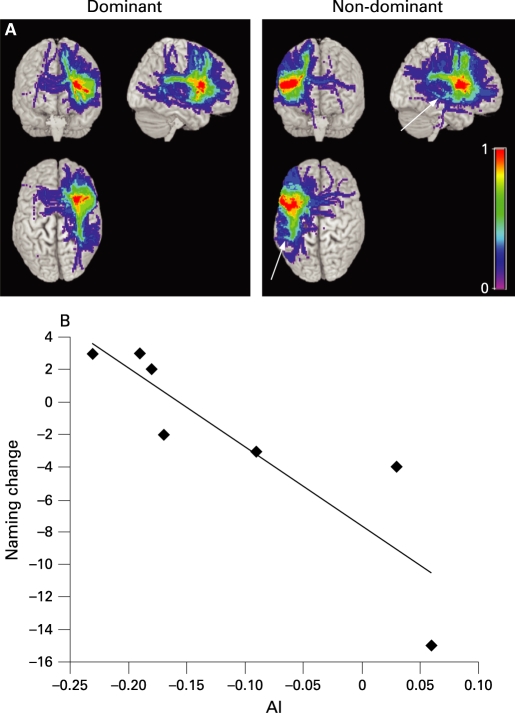
(A) Frontal lobe connections. Group variability maps of the connecting paths tracked from the dominant and non-dominant frontal regions of interest in patients undergoing anterior temporal lobe resection (ATLR) of the language dominant hemisphere. Each image shows the maximum intensity of the commonality maps in each plane of view as a brain surface rendering. The colour scale indicates the degree of overlap among subjects (expressed as commonality value); for example, a value of 1 (pure red) represents 100% subject overlap (ie, every subject’s identified tract contains the voxel in question). Greater temporal lobe and supramarginal gyrus connections were seen in the non-dominant hemisphere (arrowed). (B) Correlations between lateralisation (asymmetry index, AI) and postoperative naming change. Relationship between preoperative asymmetry of language connections and postoperative naming change. In patients undergoing dominant ATLR, a significant correlation was seen between tract lateralisation and postoperative naming decline.

Bilateral connections were demonstrated extending posteriorly from the frontal lobe ROIs. Previously, we demonstrated greater connections (particularly frontotemporal connections) via the superior longitudinal fasciculus in the left hemisphere than in the right in control subjects.^9^ Patients undergoing dominant hemisphere ATLR demonstrated less dominant and greater non-dominant hemisphere connections than controls. In particular, patients had greater temporal lobe and supramarginal gyrus connections in the non-dominant hemisphere (fig 1A).^10^

A significant correlation was demonstrated between lateralisation of connections in patients prior to dominant hemisphere ATLR and preoperative naming score (Spearman’s correlation coefficient = 0.786; p = 0.036), characterised by better preoperative naming scores in patients with connections more lateralised to the dominant hemisphere. There was also a significant correlation between lateralisation of connections and postoperative change in naming score (Spearman’s correlation coefficient = −0.991; p<0.001) (fig 1B), characterised by a greater language decline in patients with more extensive connections on the side of ATLR.

Finally, there was a significant correlation between age of onset of epilepsy and lateralisation of connections, characterised by more bilateral connections in patients with earlier age of onset (Spearman’s correlation coefficient = 0.741; p = 0.05).

## DISCUSSION

We used tractography to delineate the connections of functional language regions in TLE patients and demonstrated for the first time how preoperative knowledge of these connections may allow the prediction of language decline following ATLR. Our finding of a correlation between age of onset and lateralisation of connections is consistent with, and extends, the established theory that the earlier the occurrence of damage to the dominant hemisphere the greater the reorganisation of language function to the contralateral hemisphere.^3^ We have demonstrated a structural correlate of this functional reorganisation. The fact that patients with connections more lateralised to the dominant hemisphere had better preoperative naming scores suggests however that this structural reorganisation of language connections was not an efficient means of preserving function. Conversely, however, more bilateral connections appear to protect against postoperative language decline.

We have previously demonstrated a correlation between individuals’ degree of functional lateralisation and the lateralisation of the structural connections,^9^ ^10^ suggesting that patients with greater functional reorganisation to the non-dominant hemisphere also have an associated structural reorganisation. These results suggest that patients with relatively less reorganisation, both of function and structure, and who have greater dominant hemisphere connections, are at greater risk of language decline following ATLR in the speech dominant hemisphere. Interestingly, there was no significant correlation between the degree of language fMRI lateralisation and postoperative language change within this group. Some variability exists in the extent of patients’ temporal lobe connections and another possibility is that direct surgical disruption of temporal lobe connections leads to a decline in naming.

The largest postoperative decline in naming score was observed in the patient undergoing a right ATLR. This patient demonstrated extensive right hemisphere activation on language fMRI tasks. His pattern of neuropsychological test results, with a significant decline in verbal memory score and little change in non-verbal memory, were also in keeping with resection of the language dominant hemisphere. This patient was left-handed and presumably his language dominance was firmly established prior to the development of his glioma. He was rendered seizure free by surgery and there were no electroclinical features to suggest left hemisphere pathology. Excluding this subject from the group analyses did not significantly change the results of the correlation with postoperative language decline. For a test to be clinically useful it needs to be applicable to all patients having ATLR, regardless of aetiology and language dominance, and we therefore feel that patients with alternative pathologies and those with atypical language dominance should be included in these types of studies. We recognise however that this inevitably reduces the homogeneity of the sample and adds in other potentially confounding variables.

Our findings relate to a relatively small sample of patients. As a result, the findings and conclusions are preliminary in nature and will require confirmation in larger groups. A clinically significant decline in naming ability postoperatively was highlighted in the neuropsychological reports of two patients (Nos 5 and 7). These two also reported a decline in fluency, and an increase in word finding difficulties following surgery. It is encouraging that for both of these cases the changes in language test scores mirrored the patients’ own observation. The other patients did not describe any postoperative change in language functions. The sample size did not allow us to investigate fully the influence of other potentially important factors, for example the effect of handedness, underlying pathology and the extent and outcome of surgery on our findings.

The lower asymmetry indices observed in patients with left TLE are likely to be due to a combination of both reduced ipsilateral and greater contralateral connections. One possible explanation for the reduced ipsilateral connections is that they could be secondary to a reduction in cortical volume in the pathological language dominant hemisphere. However, all the subjects’ images were reported by neuroradiologists who did not comment on any volume loss in the frontal lobes. In addition, the ROIs selected were all in white matter underlying language cortex and therefore should be relatively unaffected by minor cortical abnormalities. Previously we also demonstrated an asymmetry of language connections in a group of healthy controls and therefore feel that on balance the asymmetries seen are not merely secondary to reduced cortical volumes in the epilepsy patients.

We demonstrated previously how tractography of the optic radiation could be used in the prediction of postoperative visual field defects occurring as a result of white matter fibre disruption.^19^ This study demonstrates a further example of how tractography may predict postoperative complications. Further prospective studies in larger populations are necessary to confirm that tractography can be used to predict postoperative language deficits. Knowing who is at risk of language impairment postoperatively would allow improved preoperative patient counselling and may guide the surgical technique to minimize such risks.
